# YGMD: a repository for yeast cooperative transcription factor sets and their target gene modules

**DOI:** 10.1093/database/bax085

**Published:** 2017-11-06

**Authors:** Wei-Sheng Wu, Pin-Han Chen, Tsung-Te Chen, Yan-Yuan Tseng

**Affiliations:** 1 Department of Electrical Engineering, National Cheng Kung University, Tainan 70101, Taiwan; 2 Center for Molecular Medicine and Genetics, Wayne State University, School of Medicine, Detroit, MI 48201, USA

## Abstract

By organizing the genome into gene modules (GMs), a living cell coordinates the activities of a set of genes to properly respond to environmental changes. The transcriptional regulation of the expression of a GM is usually carried out by a cooperative transcription factor set (CoopTFS) consisting of several cooperative transcription factors (TFs). Therefore, a database which provides CoopTFSs and their target GMs is useful for studying the cellular responses to internal or external stimuli. To address this need, here we constructed YGMD (Yeast Gene Module Database) to provide 34120 CoopTFSs, each of which consists of two to five cooperative TFs, and their target GMs. The cooperativity between TFs in a CoopTFS is suggested by physical/genetic interaction evidence or/and predicted by existing algorithms. The target GM regulated by a CoopTFS is defined as the common target genes of all the TFs in that CoopTFS. The regulatory association between any TF in a CoopTFS and any gene in the target GM is supported by experimental evidence in the literature. In YGMD, users can (i) search the GM regulated by a specific CoopTFS of interest or (ii) search all possible CoopTFSs whose target GMs contain a specific gene of interest. The biological relevance of YGMD is shown by a case study which demonstrates that YGMD can provide a GM enriched with genes known to be regulated by the query CoopTFS (Cbf1-Met4-Met32). We believe that YGMD provides a valuable resource for yeast biologists to study the transcriptional regulation of GMs.

**Database URL:** http://cosbi4.ee.ncku.edu.tw/YGMD/, http://cosbi5.ee.ncku.edu.tw/YGMD/ or http://cosbi.ee.ncku.edu.tw/YGMD/

## Introduction

In response to internal or external stimuli, a living cell would coordinately express a set of functionally related genes, termed a gene module (GM) (1). The transcriptional regulation of the spatio-temporal expression pattern of a GM is usually controlled by a cooperative transcription factor set (CoopTFS) consisting of several cooperative transcription factors (TFs) (2–4). Therefore, identifying CoopTFSs and their target GMs is important for understanding cellular responses to environmental changes.

Computational approaches have been developed to predict cooperative TF pairs (5–10) or GMs (11–15) in *Saccharomyces cerevisiae*. On the other hand, two yeast databases have been constructed by collecting TFs and their target GMs with experimental evidence from the literature. First, YEASTRACT (16) collects 307 GMs, each of which is regulated by a single TF. The regulatory associations between a TF and its target GM are supported by experimental evidence in the literature. Second, YCRD (17) collects 2535 GMs, each of which is regulated by a predicted cooperative TF pair. The regulatory associations between a predicted cooperative TF pair and its target GM are supported by experimental evidence in the literature.

Note that YEASTACT only provides GMs regulated by a single TF and YCRD only provides GMs regulated by a predicted cooperative TF pair. Considering only one or two TFs is a limitation of these two databases because biologists have demonstrated that more than two TFs could form a TF complex to co-regulate a GM. For example, Fkh2-Mcm1-Ndd1 TF complex regulates a GM expressed in the G2/M phase of the cell cycle (18). Cbf1-Met4-Met32 TF complex regulates a GM involved in the sulfur metabolism (19). Hap2-Hap3-Hap4-Hap5 TF complex regulates a GM involved in the respiratory process (20–22). Therefore, it is advantageous to have a database to provide CoopTFSs, each of which may consist of more than two cooperative TFs, and their target GMs.

To address this need, we construct YGMD (Yeast Gene Module Database) to provide 34120 GMs, each of which is regulated by a CoopTFS consisting of two to five cooperative TFs. The cooperativity between TFs in a CoopTFS is suggested by physical/genetic interaction evidence or/and predicted by existing algorithms. The target GM regulated by a CoopTFS is defined as the common target genes of all the TFs in a CoopTFS. The regulatory association between any TF in a CoopTFS and any gene in the target GM is supported by only TF binding evidence or both TFB and TF regulation evidences (see ‘Data collection’ section for details). We believe that YGMD provides a valuable resource for yeast biologists to study the underlying molecular mechanisms of cellular responses to environmental changes.

## Construction and contents

### Data collection

Seven types of data were used to construct YGMD. First, the target genes of 201 TFs (validated by TFB evidence) and the target genes of 160 TFs (validated by both TFB evidence and TFR evidence) were downloaded from YEASTRACT (16). TFB evidence is the experimental evidence (from ChIP assay, foot-printing or band-shift) showing that a TF binds to the promoters of its target genes. TFR evidence is the experimental evidence (from genome-wide expression analysis or detailed gene by gene analysis) showing that a TF perturbation (over-expression or knockout) causes a significant change in the expression of its target genes. Second, the physical and genetic interaction data of all yeast genes were downloaded from BioGRID (23). The Saccharomyces Genome Database (SGD) (24) is the best-known yeast database which provides comprehensive integrated biological information for the budding yeast *S.cerevisiae*. SGD chooses BioGRID as the source of interaction data. Following SGD, we use BioGRID as the source of interaction data in YGMD. Third, 2622 predicted cooperative TF pairs were collected from 17 existing algorithms [see CoopTFD (25) for details]. Fourth, nine kinds of associations between 695005 yeast gene-gene pairs were downloaded from YeastNet (26). The associations include co-citation, co-expression, co-occurrence of protein domains, similar genomic context of bacterial orthologs, similar profiles of genetic interaction partners, high-throughput protein–protein interactions, small/medium-scale protein–protein interactions, similar phylogenetic profiles, and 3D protein structure of interacting orthologous proteins. Finally, the last three types of data [Gene Ontology (GO) terms, literature data and biochemical pathway data] for all yeast genes were downloaded from SGD (24).

### Construction of CoopTFSs

In YGMD, we constructed CoopTFSs, each of which consists of two to five TFs. The reason for considering the number of TFs in a CoopTFS up to five is due to the computational complexity. For example, the number of possible TF sets of six TFs is $C_{6}^{201}$, which is larger than $8\times 10^{10}$. Therefore, YGMD only provides CoopTFSs consisting of five or less TFs.

**Figure 1.**
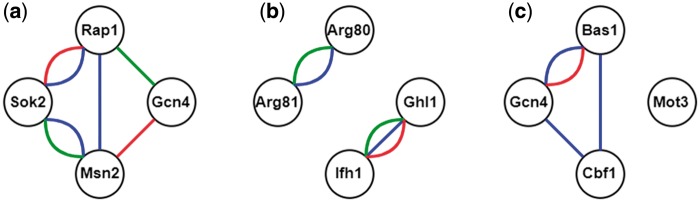
TF sets which may or may not be CoopTFSs. **(a)** The TF set {Gcn4, Msn2, Rap1, Sok2} is a CoopTFS. **(b)** The TF set {Arg80, Arg81, Ghl1, Ifh1} is not a CoopTFS. **(c)** The TF set {Bas1, Cbf1, Gcn4, Mot3} is not a CoopTFS. Red/Green lines between two TFs represent genetic/physical interactions. Blue lines between two TFs means that these two TFs have cooperativity predicted by existing algorithms.

Here we illustrate the procedure of constructing all possible CoopTFSs of four TFs as an example. First, use all the TFs in YEASTRACT (16) to enumerate all possible TF sets of four TFs. Second, construct a corresponding 4-node network for each TF set of four TFs. In this network, two nodes (i.e. TFs) are connected by an edge if they have physical interaction, genetic interaction [retrieved from BioGRID (23)] or predicted cooperativity by existing algorithms [retrieved from CoopTFD (25)]. Finally, a TF set is called a CoopTFS if the corresponding four-node network has only one connected component. That is, any two nodes in the network are connected to each other by paths. Our rationale is that any two TFs in a CoopTFS should have direct (i.e. connected by an edge) or at least indirect (i.e. connected by a path) cooperativity. The direct cooperativity between two TFs is suggested by physical/genetic interaction evidence or/and predicted by existing algorithms. Here we give three examples to clarify the concept. As shown in Figure 1, The TF set {Gcn4, Msn2, Rap1, Sok2} is a CoopTFS but the TF sets {Arg80, Arg81, Ghl1, Ifh1} and {Bas1, Cbf1, Gcn4, Mot3} are not.

The rationale of using a connected component rather than a clique to define a CoopTFS is as follows. Biologically, the components of a protein complex may not have physical interactions. For example, Fkh2-Mcm1-Ndd1 is a known TF complex which regulates genes expressed in the G2/M of the cell cycle (18). However, Mcm1 and Ndd1 do not have physical interaction. Therefore, Fkh2-Mcm1-Ndd1 forms a connected component but not a clique in a TF network whose edges represent physical interactions. Of course, there are known TF complexes [e.g. Cbf1-Met4-Met28 (19) and Hap2-Hap3-Hap4-Hap5 (20–22)] form cliques.

### Construction of the target GM of a CoopTFS

The target GM regulated by a CoopTFS is defined as the common target genes of all the TFs in a CoopTFS. Two kinds of GMs could be defined. For the first kind of GMs, the regulatory association between any TF in a CoopTFS and any gene in the target GM is supported by TFB&TFR evidence. For the second kind of GMs, the regulatory association between any TF in a CoopTFS and any gene in the target GM is only supported by TFB evidence. Note that the first kind of GMs is more biologically meaningful than the second kind because the former has stronger evidence of regulatory associations than the latter does. It also can be imagined that the number the genes in the first kind is smaller than those in the second kind. For example, the number of genes in the target GM of the CoopTFS (Cbf1-Met4-Met32) validated by TFB&TFR evidence is 16, while the number of genes in the target GM of the same CoopTFS (Cbf1-Met4-Met32) validated by TFB evidence increases to 115. The detailed statistics of the CoopTFSs in YGMD could be seen in Table 1.

**Table 1.** The detailed information of CoopTFSs in YGMD

	No. of CoopTFSs (2)^a^	No. of CoopTFSs (3)	No. of CoopTFSs (4)	No. of CoopTFSs (5)	Total no. of CoopTFSs
TFB&TFR evidence^b^	346	446	253	51	1096
TFB evidence^c^	1188	4629	10550	16657	33024

a CoopTFSs (2) means CoopTFSs of two TFs.

b Regulatory association is validated by TFB&TFR evidence. Only the CoopTFSs whose target GMs contain at least five genes are kept.

c Regulatory association is validated by TFB evidence. Only the CoopTFSs whose target GMs contain at least 15 genes are kept.

### Identification of the enriched GO terms and pathways of a GM

For each GM, YGMD provides a tool to identify the enriched GO terms and pathways. The hypergeometric distribution is used to test the statistical significance of enrichment (27). The procedures for checking whether a specific GO term is enriched in a given GM are as follows. Let *S* be the set of genes which are annotated to that specific GO term, *R* be the set of genes of a given GM, T=S∩R be the set of genes which are annotated to that specific GO term and are also in the given GM, and *F* be the set of all genes in the yeast genome. Then the *P*-value for rejecting the null hypothesis (*H*_0_: the specific GO term is not enriched in the given GM) is calculated as

$$P-value=P\left(x\geq \left|T\right|\right)=\sum_{x\geq \left|T\right|}\frac{\left(\begin{array}{c} \left|S\right|\\ x \end{array}\right)\left(\begin{array}{c} \left|F\right|-\left|S\right|\\ \left|R\right|-x \end{array}\right)}{\left(\begin{array}{c} \left|F\right|\\ \left|R\right| \end{array}\right)}$$

where $\left|S\right|$ means the number of genes in set *S*. This *P*-value is then corrected by the Bonferroni correction to represent the true alpha level in the multiple hypotheses testing. A specific GO term is said to be enriched in the given GM if the Bonferroni-corrected *P-*value is < 0.01. Note that the procedure for checking whether a specific pathway is enriched in a given GM is the same as above-mentioned procedure.

### Implementation of the web interface of YGMD

The web interface of YGMD was constructed using the PHP language with the CodeIgniter MVC framework. The information of CoopTFSs and their target GMs were deposited in MySQL. All tables and network graphs were produced by the JavaSscript and feature-rich JavaScript libraries [jQuery, DataTables and Cytoscape Web (28)] to visualize data on the webpage.

## Utility and discussion

### Database interface

**Figure 2.**
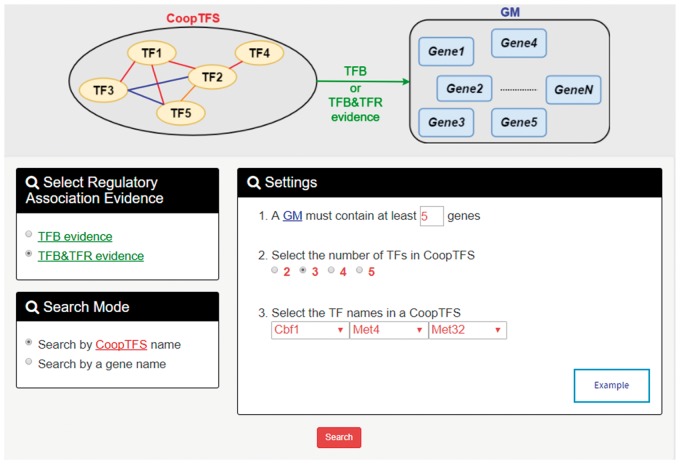
The first search mode (search by a CoopTFS name). Users have to select a CoopTFS of interest, the experimental evidence (TFB or TFB&TFR) of the regulatory associations, and a least number of genes that a GM must contain.

**Figure 3.**
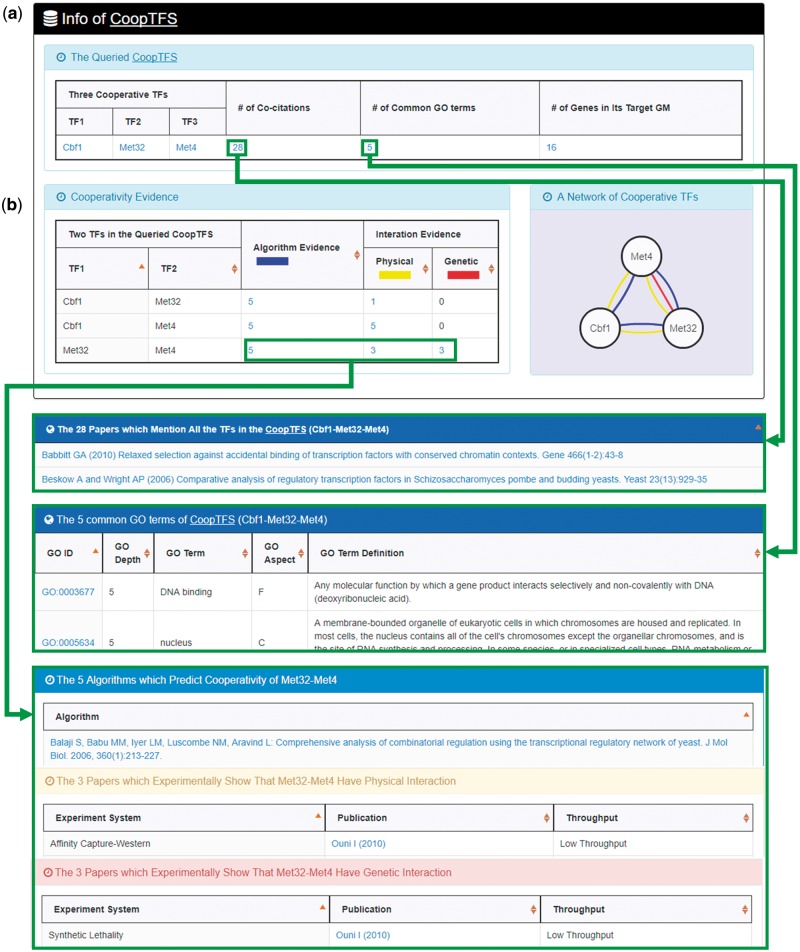
The result page of the first search mode (I). The result page consists of five parts. The first two parts are as follows. **(a)** For the chosen CoopTFS, the names of the TFs, the number of co-citations of these TFs, the number of common GO terms of these TFs, and the number of genes in its target GM are provided. **(b)** A network of cooperative TFs for the chosen CoopTFS is constructed. An edge between two TFs exists if these two TFs have physical interaction, genetic interaction or predicted cooperativity (from existing algorithms).

**Figure 4.**
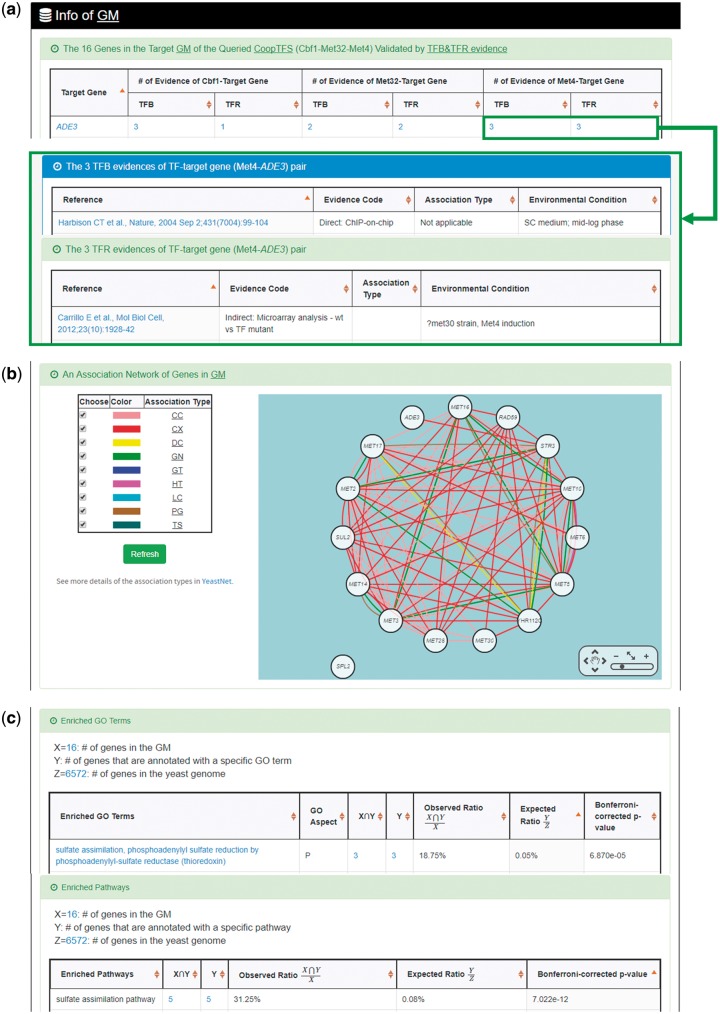
The result page of the first search mode (II). The result page consists of five parts. The last three parts are as follows. **(a)** The names of genes in the target GM and the number of experimental evidence of the regulatory association between any TF in the CoopTFS and any gene in its target GM are given. **(b)** An association network of genes in the target GM is constructed. An edge between two genes exists if these two genes have at least one of the nine kinds of associations defined by YeastNet. **(c)** The enriched GO terms and pathways of the GM are identified.

YGMD provides two search modes and three browse modes. In the first search mode (i.e. search by a CoopTFS name), users have to select a CoopTFS of interest, the experimental evidence (TFB or TFB&TFR) of the regulatory associations, and a least number of genes that a GM must contain (Figure 2). After submission, YGMD returns a result page of five parts: (i) For the chosen CoopTFS, YGMD provides the names of the TFs, the number of co-citations of these TFs, the number of common GO terms of these TFs, and the number of genes in its target GM (Figure 3a). Note that if a CoopTFS is of biological relevance, we expect to see many co-citations and common GO terms. (ii) A network of cooperative TFs for the chosen CoopTFS is constructed. An edge between two TFs exists if these two TFs have physical interaction (23), genetic interaction (23) or predicted cooperativity (25) (Figure 3b). Note that if a CoopTFS is of biological relevance, we expect to see many edges in the network. (iii) The names of genes in the target GM and the number of experimental evidence of the regulatory association between any TF in the CoopTFS and any gene in its target GM are given (Figure 4a). Note that the regulatory association of every TF-gene pair has literature evidence (16). (iv) An association network of genes in the target GM is constructed. An edge between two genes exists if these two genes have at least one of the nine kinds of associations defined by YeastNet (26) (Figure 4b). Note that if a GM is of biological relevance, we expect to see many edges in the network. (v) The enriched GO terms and pathways of the GM are identified (Figure 4c). Note that if a GM is of biological relevance, we expect to see some enriched GO terms and enriched pathways.

**Figure 5.**
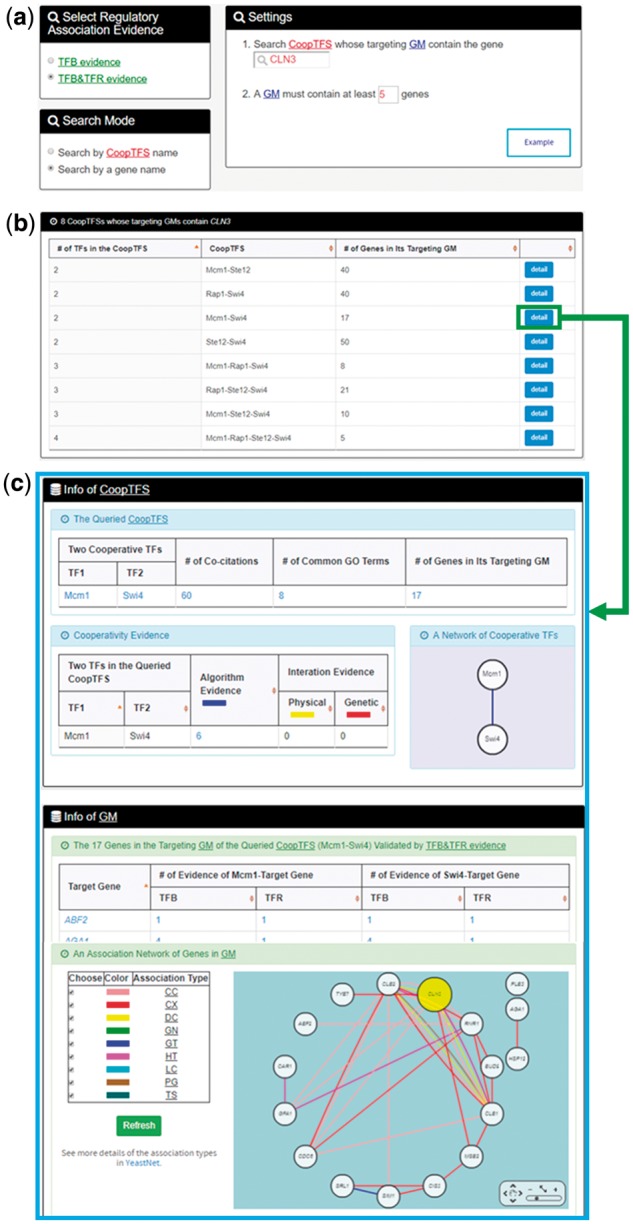
The input and output pages of the second search mode. **(a)** In the second search mode (i.e. search by a gene name), users have to select a gene of interest, the experimental evidence (TFB or TFB&TFR) of the regulatory associations, and a least number of genes that a GM must contain. **(b)** After submission, YGMD returns all possible CoopTFSs whose target GMs contain the gene of interest. **(c)** The detailed information of each CoopTFS could be found by clicking the ‘detail’ button.

In the second search mode (i.e. search by a gene name), users have to select a gene of interest, the experimental evidence (TFB or TFB&TFR) of the regulatory associations, and a least number of genes that a GM must contain (Figure 5a). After submission, YGMD returns all possible CoopTFSs whose target GMs contain the gene of interest (Figure 5b). The detailed information of each CoopTFS could be found by clicking the ‘detail’ button (Figure 5c).

**Figure 6.**
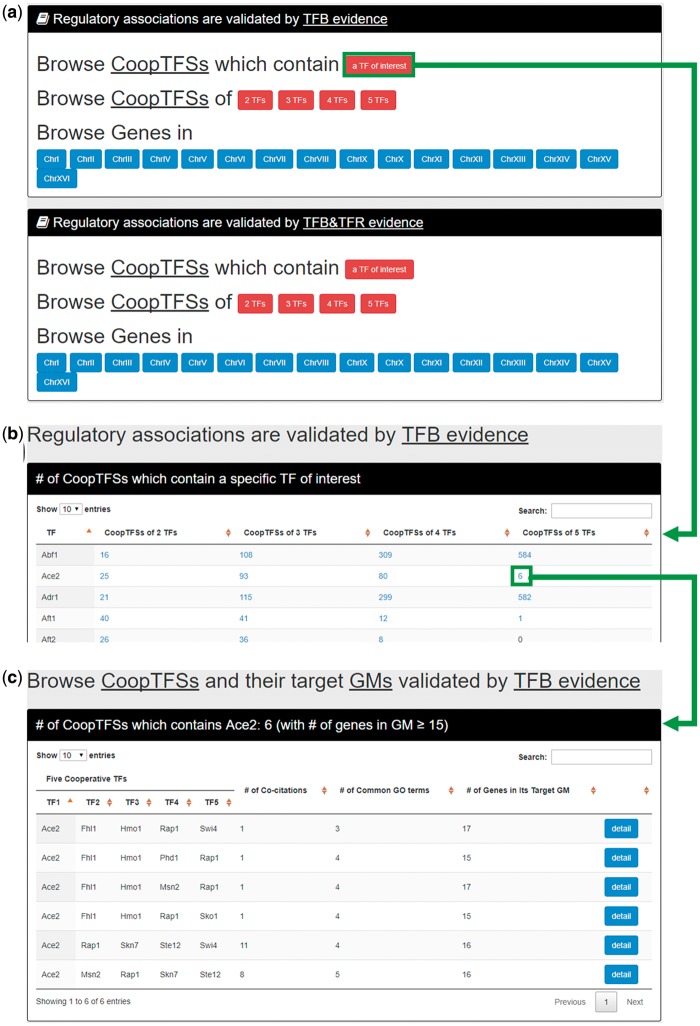
The input and output pages of the first browse mode. **(a)** In the first browse mode (i.e. browse by TFs), users have to select the experimental evidence (TFB or TFB&TFR) of the regulatory associations. **(b)** After submission, YGMD returns the number of CoopTFSs which contain a TF of interest. (c) The detailed information of the CoopTFSs could be found by clicking the number.

In the first browse mode (i.e. browse by TFs), users have to select the experimental evidence (TFB or TFB&TFR) of the regulatory associations (Figure 6a). After submission, YGMD returns the number of CoopTFSs which contain a TF of interest (Figure 6b). The detailed information of the CoopTFSs could be found by clicking the number (Figure 6c).

**Figure 7.**
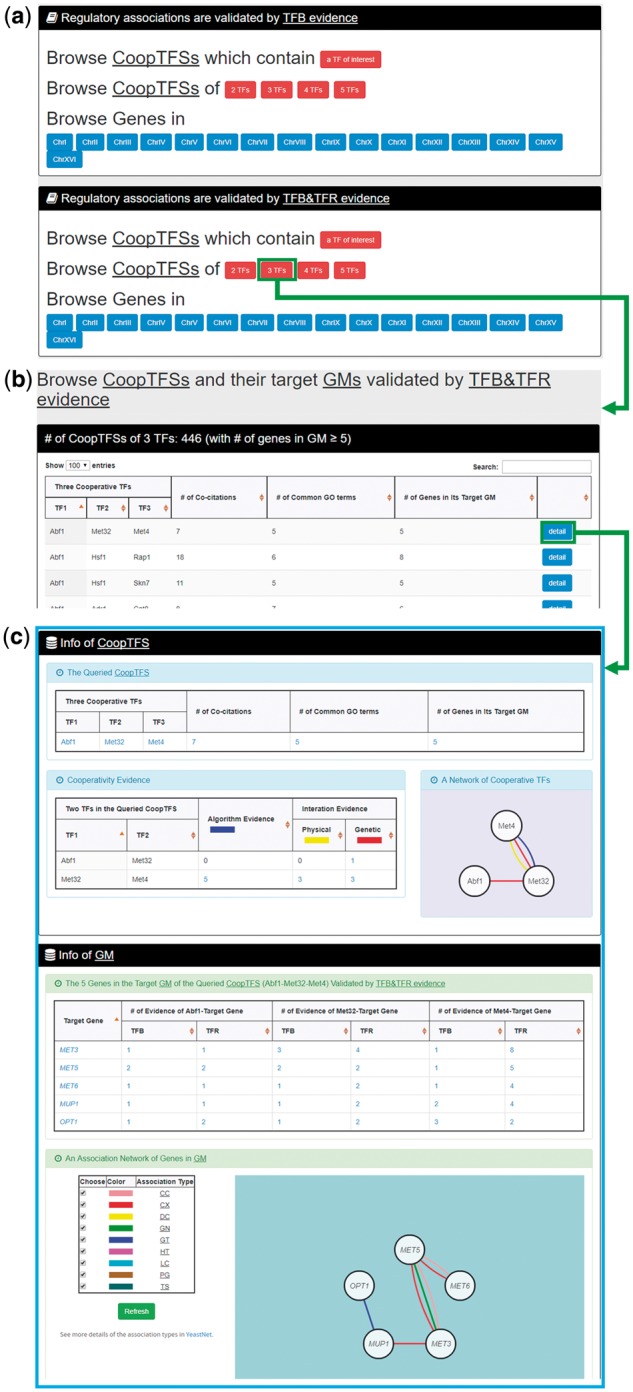
The input and output pages of the second browse mode. **(a)** In the second browse mode (i.e. browse by CoopTFSs), users have to select two settings: the number of TFs in a CoopTFS and the experimental evidence (TFB or TFB&TFR) of the regulatory associations. **(b)** After submission, YGMD returns all CoopTFSs which satisfy the settings and have at least five (for choosing TFB&TFR) or fifteen (for choosing TFB) genes in its target GM. **(c)** The detailed information of each CoopTFS could be found by clicking the ‘detail’ button.

In the second browse mode (i.e. browse by CoopTFSs), users have to select two settings: the number of TFs in a CoopTFS and the experimental evidence (TFB or TFB&TFR) of the regulatory associations (Figure 7a). After submission, YGMD returns all CoopTFSs which satisfy the settings and have at least five (for choosing TFB&TFR) or 15 (for choosing TFB) genes in its target GM (Figure 7b). The detailed information of each CoopTFS could be found by clicking the ‘detail’ button (Figure 7c).

**Figure 8.**
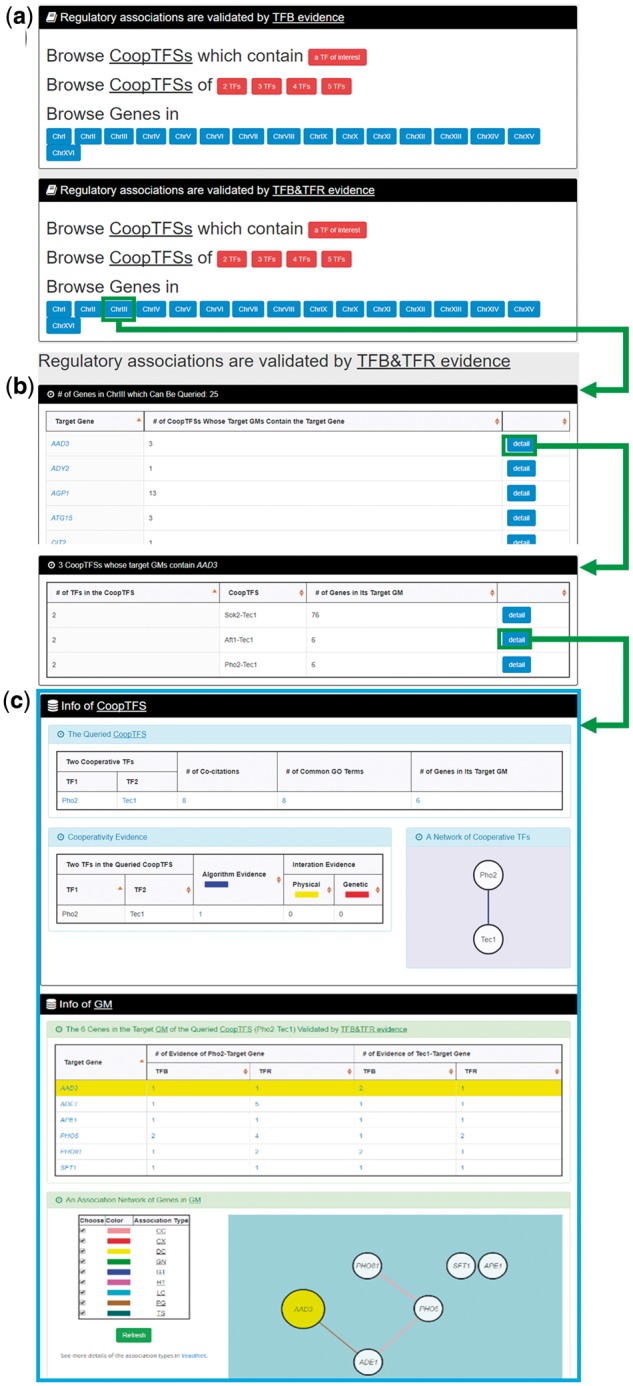
The input and output pages of the third browse mode. **(a)** In the third browse mode (i.e. browse by chromosomes), users have to select a specific chromosome of interest and the experimental evidence (TFB or TFB&TFR) of the regulatory associations. **(b)** After submission, YGMD returns all genes in that specific chromosome. For each gene, the number of all possible CoopTFSs whose target GMs contain the gene of interest is shown. **(c)** The detailed information of each CoopTFS could be found by clicking the ‘detail’ button.

In the third browse mode (i.e. browse by chromosomes), users have to select two settings: a specific chromosome of interest and the experimental evidence (TFB or TFB&TFR) of the regulatory associations (Figure 8a). After submission, YGMD returns all genes in that specific chromosome. For each gene, the number of all possible CoopTFSs whose target GMs contain the gene of interest is shown (Figure 8b). The detailed information of each CoopTFS could be found by clicking the ‘detail’ button (Figure 8c).

### A case study

**Figure 9.**
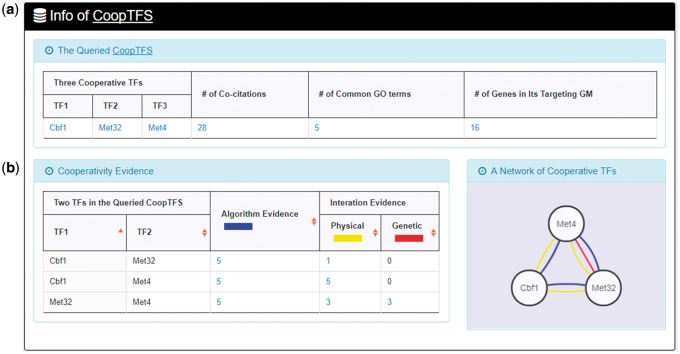
Info of CoopTFS (Cbf1-Met4-Met32). YGMD provides two kinds of information to check the biological relevance of the queried CoopTFS (Cbf1-Met4-Met32). **(a)** The three TFs Cbf1, Met4 and Met32 are co-appearance in 28 publications and have 5 common GO terms, suggesting that they may form a CoopTFS to regulate the expressions of a set of genes. **(b)** Cbf1-Met4, Cbf1-Met32 and Met4-Met32 all have protein–protein interactions, indicating that Cbf1-Met4-Met32 can really form a TF complex to regulate genes’ expressions.

Here we use a case study to demonstrate that YGMD can provide biologically meaningful results for users’ query. Cbf1-Met4-Met32 is a well-known TF complex which transcriptionally regulates a set of genes involved in sulfur amino acid biosynthesis pathway (19). If we query the CoopTFS (Cbf1-Met4-Met32) in YGMD (Figure 2), the result page is shown in Figures 3 and 4. In the result page, YGMD provides two kinds of information to check the biological relevance of the queried CoopTFS (Cbf1-Met4-Met32). First, the three TFs Cbf1, Met4 and Met32 are co-appearance in 28 publications and have 5 common GO terms (Figure 9a), suggesting that they may form a CoopTFS to regulate the expressions of a set of genes. Second, Cbf1-Met4, Cbf1-Met32 and Met4-Met32 all have protein–protein interactions (Figure 9b), indicating that Cbf1-Met4-Met32 can really form a TF complex to regulate genes’ expressions.

**Figure 10.**
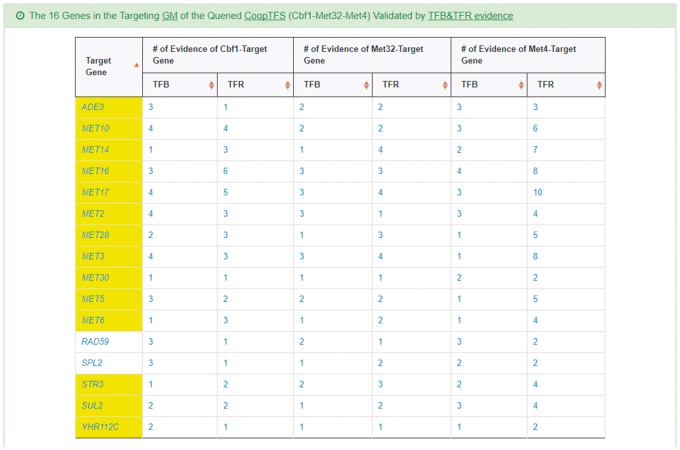
The target GM of CoopTFS (Cbf1-Met4-Met32). The target GM for the query CoopTFS (Cbf1- Met4-Met32) contains 16 genes. The regulatory association between any TF in a CoopTFS and any gene in the target GM is supported by both TFB&TFR evidence. Note that the column of ‘Target Gene’ is colored yellow if the gene is involved in sulfur metabolism. See more details at http://cosbi4.ee.ncku.edu.tw/YGMD/sulfur_cbf1_met4_met32_TFBR.

**Figure 11.**
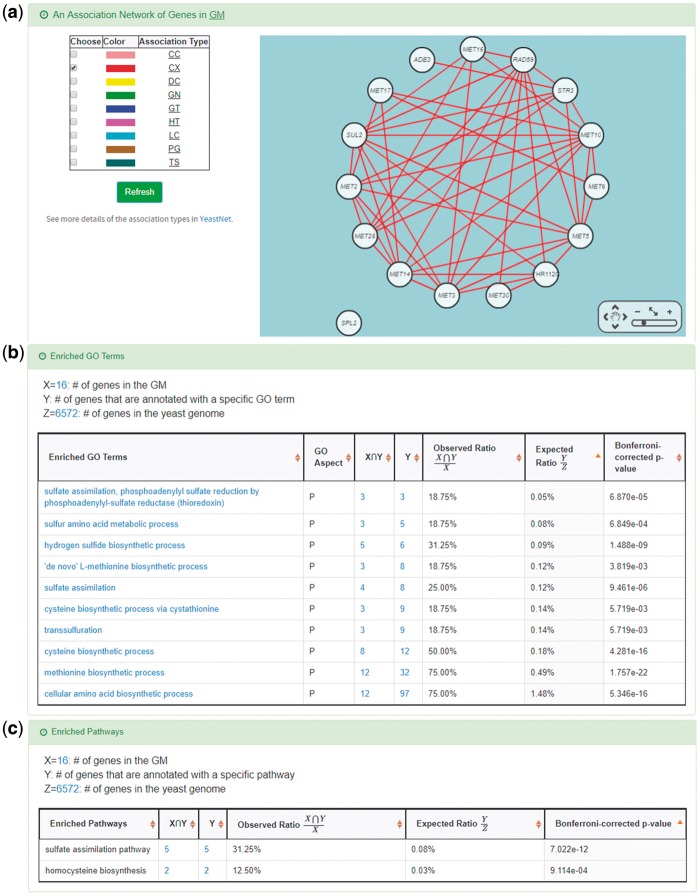
Info of the target GM of CoopTFS (Cbf1-Met4-Met32). YGMD provides three kinds of information to check the biological relevance of the GM. **(a)** The genes in the GM form a dense co-expression (Association Type: CX) network, suggesting that they may be co-regulated. **(b)** 10 enriched GO terms are identified. All of them are related to sulfur metabolism, suggesting that the GM is possibly to be regulated by the query CoopTFS (Cbf1-Met4-Met32). **(c)** Two enriched pathways are identified. Both of them are related to sulfur metabolism, suggesting that the GM is possibly to be regulated by the query CoopTFS (Cbf1-Met4-Met32).

YGMD also provides the target GM for the query CoopTFS (Cbf1-Met4-Met32). The target GM contains 16 genes (Figure 10). The regulatory association between any TF in a CoopTFS and any gene in the target GM is supported by both TFB&TFR evidence. For example, 3 TFB evidences show that TF Cbf1 binds to the promoter of gene *ADE3* and 1 TFR evidence shows that the perturbation of TF Cbf1 causes a significant change in the expression of gene *ADE3* (Figure 10). Moreover, YGMD provides three kinds of information to check the biological relevance of the GM. First, the genes in the GM form a dense co-expression network (Figure 11a), suggesting that they may be co-regulated. Second, 10 enriched GO terms are identified (Figure 11b). All of them are related to sulfur metabolism, suggesting that the GM is possibly to be regulated by the query CoopTFS (Cbf1-Met4-Met32). Third, two enriched pathways are identified (Figure 11c). Both of them are related to sulfur metabolism, suggesting that the GM is possibly to be regulated by the query CoopTFS (Cbf1-Met4-Met32).

**Figure 12.**
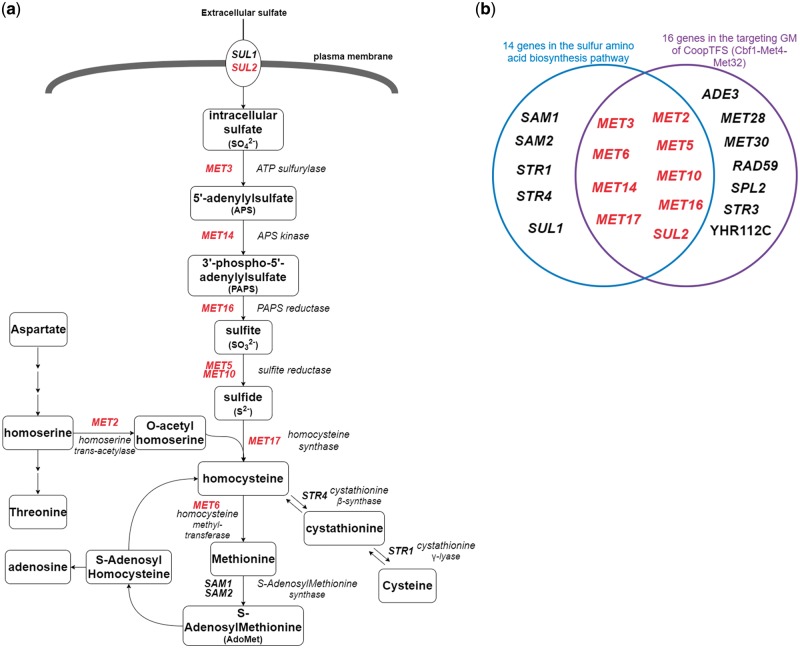
The sulfur amino acid biosynthesis pathway. **(a)** 14 genes involved in the sulfur amino acid biosynthesis pathway are shown. Gene names are colored red if they are in the target GM of the CoopTFS (Cbf1-Met4-Met32). **(b)** Since Cbf1-Met4-Met32 is known to regulate genes in sulfur amino acid biosynthesis pathway, we test the overlap between the set of genes in the sulfur amino acid biosynthesis pathway and the set of genes in the GM. Strikingly, the overlap between these two sets of genes is statistically significant (*P*-value = 3.6e-22 using the hypergeometric testing).

Finally, since Cbf1-Met4-Met32 is known to regulate genes in sulfur amino acid biosynthesis pathway (19, 29) (Figure 12a), we test the overlap between the set of genes in the sulfur amino acid biosynthesis pathway (29) and the set of genes in the GM (Figure 12b). Strikingly, the overlap between these two sets of genes is statistically significant [*P-*value = 3.6e-22 using the hypergeometric testing (27)]. In summary, all these analyses together strongly demonstrate that YGMD can provide biologically relevant information for both the queried CoopTFS and its target GM.

### Comparison with our previous databases

In the past 6 years, our group has published three databases to help yeast biologists study transcriptional regulation of gene expression. First, Yeast Promoter Atlas (YPA) (30) integrates nine kinds of promoter features for each yeast gene. Second, Cooperative Transcription Factors Database (CoopTFD) (25) has a comprehensive collection of 2622 predicted cooperative TF pairs in yeast from 17 existing algorithms. Third, Yeast Combinatorial Regulation Database (YCRD) (17) deposits 434197 regulatory associations between 2535 cooperative TF pairs and 6243 genes. In this study, we present YGMD to provide 34 120 CoopTFSs, each of which consists of two to five cooperative TFs, and their target GMs. YGMD have three unique features that cannot be found in our previous databases. First, YGMD provides CoopTFSs, each of which consists of two to five cooperative TFs, whereas CoopTFD and YCRD only consider cooperative TF pairs and YPA only considers a single TF at a time. Second, YGMD provides an association network of genes in the target GM. A highly connected association network suggests the biological relevance of the target GM. Third, YGMD provides GO term and pathway enrichment analyses. Identification of enriched GO terms and pathways suggests the biological relevance of the target GM.

## Conclusion

In this study, we constructed YGMD which provides 34 120 CoopTFSs, each of which consists of two to five cooperative TFs, and their target GMs. The biological relevance of YGMD is shown by a case study which demonstrates that for the query CoopTFS (Cbf1-Met4-Met32), a key TF complex which transcriptionally regulates genes involved in the sulfur metabolism, YGMD can provide the target GM which is enriched with known structural genes required for the biosynthesis of sulfur amino acids. In the future, we plan to improve YGMD as follows. First, we will keep updating our database once updated data in SGD, YEASTRACT, BioGRID, CoopTFD and YeastNet are available. Second, we will add more enrichment analyses (e.g. identifying enriched mutant phenotypes, enriched domains, enriched post-translational modifications and enriched literature topics) on the target GMs. We believe that YGMD provides a valuable resource for yeast biologists to study the transcriptional regulation of GMs.
